# Indication and adverse event profiles of denosumab and zoledronic acid: based on U.S. FDA adverse event reporting system (FAERS)

**DOI:** 10.3389/fphar.2023.1225919

**Published:** 2023-11-01

**Authors:** Si Su, Liuqing Wu, Guibao Zhou, Lingling Peng, Huanzhe Zhao, Xiao Wang, Kuan Li

**Affiliations:** ^1^ School of Pharmacy, Guangdong Medical University, Zhanjiang, China; ^2^ Department of Pharmacy, Shenzhen People’s Hospital (The Second Clinical Medical College, Jinan University; The First Affiliated Hospital, Southern University of Science and Technology), Shenzhen, Guangdong, China; ^3^ Longgang Central Hospital of Shenzhen, Shenzhen, Guangdong, China

**Keywords:** denosumab, zoledronic acid, adverse events, off-label use, pharmacovigilance

## Abstract

**Objective:** To investigate adverse events (AEs) associated with denosumab (Dmab) and zoledronic acid (ZA), compare their association strengths, and explore potential applications to provide clinical reference.

**Methods:** We collected data from FAERS from January 2004 to November 2022 and mined AE signals for Dmab and ZA using ROR values. We compared signal intensity for same AEs and investigated off-label use. We also examined their AEs in adjuvant therapy for breast and prostate cancer.

**Results:** 154,735 reports of primary suspect drugs were analyzed in the FAERS database (Dmab: 117,857; ZA: 36,878). Dmab and ZA had 333 and 1,379 AE signals, with 189 overlaps. The AEs of Dmab included death (ROR:3.478), osteonecrosis of jaw (ROR:53.025), back pain (ROR:2.432), tooth disorder (ROR:16.18), bone pain (ROR:6.523). For ZA, the AEs included osteonecrosis (ROR:104.866), death (ROR: 3.645), pain (ROR:3.963), osteonecrosis of jaw (ROR: 91.744), tooth extraction (ROR: 142.143). Among overlap signals, Dmab showed higher strength in exostosis of the jaw (ROR: 182.66 vs. 5.769), atypical fractures (ROR: 55.589 vs. 9.123), and atypical femur fractures (ROR:49.824 vs. 4.968). And ZA exhibited stronger associations in abscess jaw (ROR: 84.119 vs. 11.12), gingival ulceration (ROR: 74.125 vs. 4.827), increased bone formation (ROR: 69.344 vs. 3.218). Additionally, we identified 528 off-label uses for Dmab and 206 for ZA, with Dmab mainly used in prostate cancer (1.04%), breast cancer (1.03%), and arthritis (0.42%), while ZA in breast cancer (3.21%), prostate cancer (2.48%), and neoplasm malignant (0.52%). For Dmab in breast cancer treatment, AEs included death (11.6%), disease progression (3.3%), and neutropenia (2.7%), while for ZA included death (19.8%), emotional disorder (12.9%), osteomyelitis (11.7%). For prostate cancer treatment, Dmab`s AEs were death (8.9%), prostate cancer metastatic (1.6%), renal impairment (1.7%), while ZA`s included death (34.4%), general physical health deterioration (19.9%), and hemoglobin decreased (18.9%).

**Conclusion:** Our analysis of FAERS database provided postmarketing surveillance data and revealed different strengths of reported AE signals between Dmab and ZA in some of their common AEs. It’s also worth noting that both drugs have potential off-label applications, which could introduce new AEs. This highlights the necessity for safety monitoring when using Dmab and ZA off-label.

## 1 Introduction

Denosumab (Dmab), the first and only one receptor activator of NF-κB ligand (RANKL) inhibitor so far, was approved for marketing by the U.S. Food and Drug Administration (FDA) in 2010 and Zoledronic acid (ZA) approved in 2001. They have similar efficacy such as applying for prevention and treatment of osteoporosis in postmenopausal females, osteoporosis in males, glucocorticoid-induced osteoporosis, hypercalcemia of malignancy, and preventing skeletal-related events (SREs) secondary to solid tumors metastases (Greear and Bankole, 2022; Hildebrand et al., 2022). However the mechanism differs between the two (Baron et al., 2011), with Dmab exerting its anti-bone resorption effect by attaching to RANKL which activate osteoclasts through the binding with RANK, thereby suppressing bone resorption (Jamal et al., 2011; Pang et al., 2020). Zoledronic acid, on the other hand, binding of inorganic pyrophosphate to hydroxyapatite crystals in bone, especially in the sites where bone is remodeling actively, and thus play an anti-bone resorption role (Drake et al., 2008). Dmab and ZA have two different drug specifications each. Dmab is available as Xgeva (120 mg) for preventing bone-related events in cancer patients and Prolia (60 mg) for treating osteoporosis. Similarly, ZA has two different specifications; Reclast (5 mg) for treating osteoporosis and Zometa (4 mg) for cancer-related bone damage.

In the past decade, significant efficacy of both drugs has been extensively documented, whereas, novel AEs not well studied were gradually raised during the clinical application. Furthermore, novel mechanisms as well as application also emerged. We hope this analysis based on FAERS database will provide safety profile in support of future studies in the application of Dmab and ZA. And to provide reference directions for exploring their potential clinical applications.

## 2 Materials and methods

### 2.1 Data sources and procedures

The data for this retrospective pharmacovigilance study were obtained from FAERS, a global spontaneous reporting system that collects safety information on approved drugs and therapeutic biologic products from various sources including manufacturers, healthcare professionals, and consumers. FAERS is the primary source of post-marketing safety monitoring and evaluation for the FDA, and it provides signal detection and quantification of the association between drugs and AEs (Tang et al., 2022). The database contains seven categories of data including demographic and management information, drug information, adverse events, patient outcomes, report sources, treatment start and end dates, and indication.

### 2.2 Data extraction and processing

To extract adverse event (AE) reports from the FDA Open-FDA program, we utilized the online tool OpenVigil 2.1 (http://openvigil.sourceforge.net/). Individual safety reports (ISRs) for Dmab and ZA were extracted from the FAERS database. ISRs are the count of raw data extracted by OpenVigil 2.1 and an ISR code represents an AE report.

The study retrieved data from FAERS covering the period between January 2004 and November 2022. The search for Dmab included its generic name “DENOSUMAB” and commodity names “Xgeva,” “Ranmark,” and “Prolia,” while for ZA, the search included its generic drug name “ZOLEDRONIC ACID,” “ZOLEDRONATE,” and trade names “ACLASTA,” “RECLAST”, and “ZOMETA.” Drugs irrelevant to the study and those with uncertain names were excluded. Only drugs listed as the “primary suspect” were included in the analysis as they were most likely associated with the AEs (Verden et al., 2018; Omar et al., 2021).

### 2.3 AE signals detection

Disproportionality analysis was conducted to identify potential safety signals for the drugs, with RORs as measures of association (van Puijenbroek et al., 2002; Hauben, 2003; Tang et al., 2022). The analysis of the association between drug exposure and adverse events (referred to as “signals”) in OpenVigil relies on the use of a 2 × 2 contingency table (Böhm, 2018; Noguchi et al., 2021) (Refer to Table 1), which can be effortlessly generated within the platform. The higher the ROR values, the stronger the correlation between the drug and target AE. Significant signals were identified based on criteria including AE reports >3, ROR and PRR >2.0, ROR lower bound of 95% confidence interval (CI) value exceeds 1.0, and *χ* ^*2*^ > 4 (Böhm, 2018; Shao et al., 2021; Tang et al., 2022). The equations and criteria for the three algorithms are shown in Table 2. Data processing was carried out using Microsoft Excel 2016 and GraphPad Prism 9.

**TABLE 1:** Two-by-two contingency table.

	Drug exposure	No drug exposure	Sums
Adverse event occurred	DE	dE	E
No adverse event occurred	De	de	e
Sums	D	d	N

**Note:** D represents occurrence of drug exposure and E represents adverse event of interest, d represents no drug exposure and e represents no occurrence of the adverse event.

**TABLE 2:** Equation and criteria of three algorithms for signal detection.

Algorithms	Equation	Criteria
ROR	ROR = (DE/De)/(dE/de)	ROR ≥ 2
	95%CI = $e^{In\left(ROR\right)\pm 1.96\sqrt{\frac{1}{DE}+\frac{1}{De}+\frac{1}{dE}+\frac{1}{de}}}$	95%CI > 1
		DE ≥ 3
PRR	PRR = (DE/D)/(dE/d)	PRR ≥ 2
		DE ≥ 3
χ^2^	χ^2^_Yates_ = N * (| DE*de – dE*De | - N/2 )^2^/(D * d * E * e)	χ^2^ ≥ 4

**Note:** ROR, reporting odds ratio; PRR, proportional reporting ratio; CI: confidence interval; χ^2^, chi-squared; DE, number of co-occurrences.

## 3 Results

### 3.1 AE reports and clinical information

**FIGURE 1:**
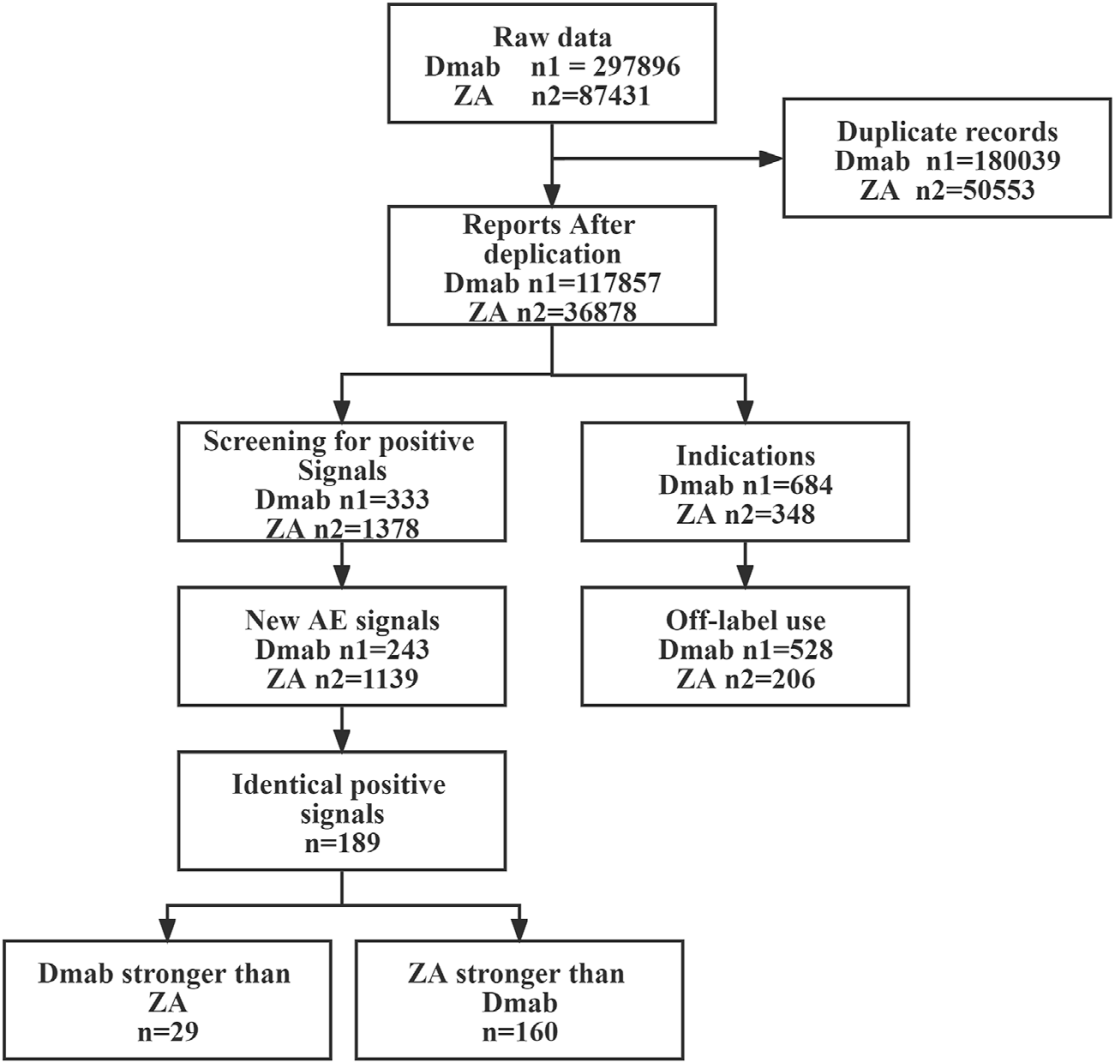
Flowchart of data mining process.

The FAERS database contained 385,327 reports of primary suspect drugs from its inception until October 2022, with 297,896 AEs associated with Dmab and 87,431 AEs related to ZA. After removing duplicates, a total of 154,735 reports were included, consisting of 117,857 AEs for Dmab and 36,878 AEs for ZA. Process flowchart is shown in Figure 1.

The characteristics and clinical information are summarized in Table 3. The majority of the reports for both drugs were from females (76.19% for Dmab and 67.09% for ZA), and the median age of the reports was 68 and 73 years for Dmab and ZA, respectively, with a focus on the elderly population.

**TABLE 3:** Characteristics and clinical information.

Characteristics	Reports (*N*, %)	
	Denosumab (n = 117,857)	Zoledronic acid (n = 36,878)
**Gender**		
Female	89,799 (76.19)	24,743 (67.09)
Male	14,136 (11.99)	10,083 (27.34)
Unknow	13,921 (11.81)	2052 (5.56)
**Age**		
Median (IQR)	68 (59–77)	73 (65–81)
<18	176 (0.15)	47 (0.13)
18–40	789 (0.67)	427 (1.16)
41–65	16,929 (14.36)	7189 (19.49)
>65	48,510 (41.16)	9847 (26.70)
Unknow	51,452 (43.66)	19,368 (52.52)
**Report countries**		
United States	88,883 (75.42)	12,431 (33.71)
Canada	6223 (5.28)	3894 (10.56)
others	22,667 (19.23)	16,109 (43.62)
Unknow	83 (0.07)	4444 (12.05)

**FIGURE 2:**
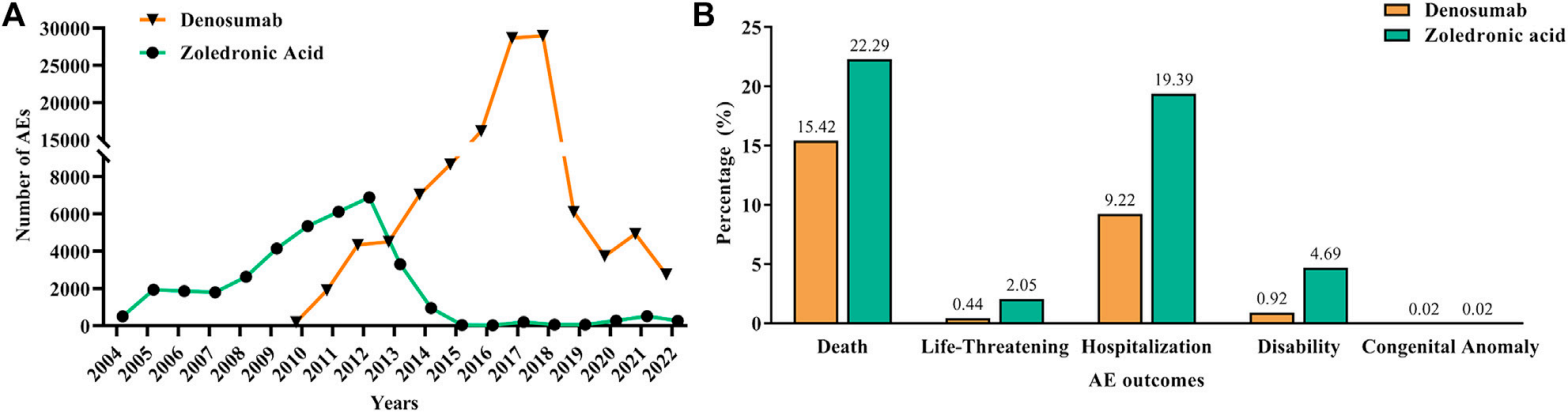
Report years and serious AE outcome information of denosumab and zoledronic acid. **(A)**: Number of reported AEs of denosumab and zoledronic acid from 2004 to 2022. **(B)**: The serious AE outcome indicators of denosumab and zoledronic acid.

In order to make the changes more intuitive, we visualized the AEs metric data of each year with a line chart, as shown in Figure 2A. The chart shows an increasing trend in AEs for both drugs year by year, but a decline in 2012 and 2018 for ZA and Dmab, respectively. In addition, we also visualized the serious AE outcome metric data for the two drugs, as shown in Figure 2B. Serious AEs were mainly attributed to death (15.42% for Dmab and 22.29% for ZA) and hospitalization (9.22% for Dmab and 19.39% for ZA). Furthermore, ZA had slightly higher proportions of life-threatening (2.05% vs. 0.44%) and disability (4.69% vs. 0.92%) according to the reports from the database compared to Dmab.

### 3.2 Differences of overall AE signals between dmab and ZA

We then conducted a disproportionality analysis using ROR to detect AE signals, which led to the identification of 333 significant AE signals related to Dmab and 1379 associated with ZA. Interestingly, 243 new AE signals and 528 off-label use for Dmab that were not registered in the FDA-approved specification were found, along with 1139 new signals and 206 off-label uses for ZA.

The most common AEs associated with Dmab were death, osteonecrosis of the jaw, back pain, tooth disorder, bone pain, and hypocalcemia. For ZA, the most frequent adverse events were osteonecrosis, death, pain, osteonecrosis of the jaw, and tooth extraction. Among these, death and fall were not mentioned in the drug labels for either Dmab or ZA. Top l0 significant AE signals sorted by frequency for both drugs are presented in Table 4.

**TABLE 4:** Top 10 significant AE signals of Dmab and ZA.

	**AEs**	**N**	**ROR (95% CI)**	**PRR (χ2)**
**Denosumab**	Death*	16,013	3.478 (3.42)	3.142 (23,699.174)
	Osteonecrosis of jaw	6043	53.025 (51.377)	50.358 (193,469.11)
	Back pain	2793	2.432 (2.342)	2.398 (2244.037)
	Tooth disorder	1929	16.18 (15.414)	15.931 (23,239.1)
	Bone pain	1878	6.523 (6.223)	6.435 (8106.468)
	Hypocalcaemia	1805	23.173 (22.009)	22.834 (30,578.129)
	Spinal fracture	1369	15.963 (15.073)	15.79 (16,337.329)
	Pain in jaw	1314	9.335 (8.819)	9.242 (8831.068)
	Fracture	894	6.768 (6.323)	6.725 (4076.903)
	Tooth extraction	815	11.487 (10.681)	11.415 (6932.845)
**Zoledronic acid**	Osteonecrosis	6980	104.866 (101.892)	85.207 (458,676.62)
	Death*	5283	3.645 (3.539)	3.266 (8597.053)
	Pain	4177	3.963 (3.836)	3.627 (8110.249)
	Osteonecrosis of jaw	3696	91.744 (88.342)	82.649 (236,587.42)
	Tooth extraction	2321	142.143 (135.258)	133.26 (214,317.12)
	Bone disorder	2129	68.221 (65.038)	64.34 (110,354.49)
	Pyrexia	1957	3.932 (3.756)	3.777 (4002.168)
	Arthralgia	1895	3.364 (3.211)	3.242 (2953.963)
	Pain in jaw	1792	44.097 (41.93)	42.002 (63,354.395)
	Fall*	1543	3.039 (2.887)	2.953 (2001.728)

**Note:** *, The instruction does not mention; 95% CI: only show the low bound of ROR.

As there were numerous shared AEs between Dmab and ZA, we conducted a further comparison of the overlapping AE signals. Out of the 189 identical positive AE signals between the two drugs, 29 AEs of Dmab exhibited stronger correlation than ZA, while 160 AEs of Dmab had weaker correlation than ZA, as determined by the ROR value. Table 5 presents the AE signals with significant differences in intensity between the two drugs. The AE signals of Dmab with stronger correlation than ZA (*d > 20*) included exostosis of jaw (ROR: 182.66 vs. 5.769), atypical fracture (ROR: 55.589 vs. 9.123), and atypical femur fracture (ROR: 49.824 vs. 4.968), while the AE signals of ZA with stronger correlation than Dmab (*d > 50*) were related to abscess jaw (ROR: 84.119 vs. 11.12), gingival ulceration (ROR: 74.125 vs. 4.827), increased bone formation (ROR: 69.344 vs. 3.218), and bone disorder (ROR: 68.221 vs. 3.189), among others.

**TABLE 5:** AE signals with significant differences in ROR values between Dmab and ZA.

Item	AEs	Dmab	ZA
		ROR (95%CI)	ROR (95%CI)
**AE signals of Dmab stronger than ZA (*d>20*)**	exostosis of jaw	182.66 (138.708)	5.769 (2.146)
	atypical fracture	55.589 (43.652)	9.123 (4.517)
	atypical femur fracture	49.824 (44.731)	4.968 (3.29)
	dental care	66.203 (58.259)	22.846 (17.792)
	dental implantation	49.816 (42.49)	22.907 (16.979)
	bone density abnormal	23.178 (20.97)	2.812 (1.848)
**AE signals of ZA stronger than Dmab (*d>50*)**	abscess jaw	11.12 (8.861)	84.119 (71.047)
	gingival ulceration	4.827 (3.115)	74.125 (58.52)
	bone formation increased	3.218 (1.427)	69.344 (47.835)
	bone disorder	3.189 (2.884)	68.221 (65.038)
	gingival erosion	4.065 (1.909)	67.58 (45.849)
	gingival erythema	2.791 (1.572)	65.051 (50.643)
	dental fistula	5.062 (3.152)	66.826 (51.022)
	periodontitis	4.408 (3.463)	62.003 (54.294)
	oroantral fistula	3.718 (1.522)	60.457 (38.31)
	bone callus excessive	11.324 (4.864)	65.817 (33.298)
	bone scan abnormal	5.185 (3.029)	59.205 (42.901)
	osteopetrosis	8.412 (3.654)	59.239 (31.974)

**Note:** *d*, difference of ROR, between denosumab zoledronic acid; 95% CI, only show the low bound of ROR.

### 3.3 Off-label use

While analyzing the data, we found off-label use was also a significant signal. Therefore, we further analyzed the data on off-label use. As we observed mixed reports of different specifications for each drug, for example, Dmab had a 60 mg specification for giant cell tumor and hypercalcemia of malignancy, while the 120 mg specification was used for postmenopausal osteoporosis. Similarly, ZA had two different specifications with mixed reports. Therefore, we combined the FDA-approved indications for both specifications of each drug and compared them with the indications in the database to identify off-label uses. We found 528 types of off-label use for Dmab and 206 types for ZA. Table 6 shows the top 10 off-label uses not mentioned in the drug instructions for both drugs, which are frequently used for treating various tumors. Breast cancer (1.03% and 3.21%) and prostate cancer (1.04% and 2.48%) were the most commonly off-label use for both drugs in the database. Other off-label uses for Dmab included arthritis (0.42%), vitamin D deficiency (0.26%), spinal compression fracture (0.25%), gastroesophageal reflux disease (0.24%), plasma cell myeloma (0.19%), rheumatoid arthritis (0.19%), and chronic kidney disease (0.10%). Off-label uses for ZA included neoplasm malignant (0.52%), renal cancer (0.45%), lung cancer (0.40%), plasma cell myeloma (0.30%), plasma cytoma (0.19%), colon cancer (0.08%), and osteoarthritis (0.07%).

**TABLE 6:** Top 10 off-label uses not mentioned in the drug instructions.

Denosumab (n = 117,857)		Zoledronic acid (n = 36,878)	
Indication	N (%)	Indication	N (%)
Prostate cancer	1225 (1.04)	Breast cancer	1184 (3.21)
Breast cancer	1215 (1.03)	Prostate cancer	916 (2.48)
Arthritis	499 (0.42)	Neoplasm malignant	190 (0.52)
Vitamin d deficiency	303 (0.26)	Renal cancer	166 (0.45)
Spinal compression fracture	299 (0.25)	Lung cancer	147 (0.40)
gastroesophageal reflux disease	286 (0.24)	Plasma cell myeloma	111 (0.30)
Plasma cell myeloma	224 (0.19)	Plasma cytoma	69 (0.19)
Rheumatoid arthritis	221 (0.19)	Colon cancer	28 (0.08)
Other solid tumors*	640 (0.54)	Osteoarthritis	25 (0.07)
Chronic kidney disease	116 (0.10)	Other solid tumors*	280 (0.76)

**Note:** *, refers to a group of tumors that includes non-small cell lung cancer, bronchial carcinoma, gastric cancer, rectal cancer, lymphoma, and more.

We conducted a comparison of the AEs associated with Dmab and ZA in off-label use for breast cancer and prostate cancer. After comparing with the FDA-approved instructions and removing similar AEs, we found 451 AEs in Dmab and 848 AEs in ZA for breast cancer treatment. For prostate cancer treatment, we found 341 AEs in Dmab and 583 AEs in ZA that were not mentioned in the drug instructions.

In breast cancer treatment, the top AEs associated with Dmab were death (11.6%), disease progression (3.3%), breast cancer metastatic (2.7%), neutropenia (2.7%), emotional disorder (2.3%), and pyrexia (1.7%). For ZA, the most frequent AEs were death (19.8%), emotional disorder (12.9%), osteomyelitis (11.4%), neoplasm progression (10.7%), cardiac disorders (10.6%), and impaired healing (9.5%). Cardiac disorders in patients with breast cancer treated with ZA included tachycardia (3.04%), congestive heart failure (1.35%), arrhythmia (1.01%), and palpitations (1.01%). Both drugs were associated with varying degrees of mental illness such as emotional distress, depression, and personality disorder, particularly in treating breast cancer, even leading to suicidal ideation. Tables 7, 8 display the six most frequent AEs in breast cancer and prostate cancer treatments, respectively, which were not registered in the drug specifications.

**TABLE 7:** Top 6 AEs not registered in specification related to breast cancer treatment.

Indication	Denosumab (n = 1215)		Zoledronic acid (n = 1184)	
	AEs	N (%)	AEs	N (%)
**Breast cancer**	Death	141 (11.6)	Death	235 (19.8)
	Disease progression	40 (3.3)	Emotional disorder ^**b**^	153 (12.9)
	Breast cancer metastatic	33 (2.7)	Osteomyelitis	135 (11.4)
	Neutropenia	33 (2.7)	Neoplasm progression	127 (10.7)
	Emotional disorder ^**a**^	28 (2.3)	Cardiac disorders ^**c**^	125 (10.6)
	Pyrexia	21 (1.7)	Impaired healing	112 (9.5)

**Note: a**. Emotional disorder in patients with breast cancer treated with Dmab included confusional state (0.74%), delirium (0.33%), disturbance in attention (0.25%), palpitations (0.25%), irritability (0.16%), anxiety (0.16%), depressed mood (0.08%), hallucination visual (0.08%), major depression (0.08%), paranoia (0.08%) and psychotic disorder (0.08%). **b**. Emotional disorder in patients with breast cancer treated with ZA, included emotional distress (3.04%), confusional state (2.53%), depression (1.86%), depressed mood (2.20%), suicidal ideation (0.84%), personality disorder (0.68%), amnesia (0.59%), abasia (0.42%), mental disorder (0.17%), emotional disorder (0.17%), disturbance in attention (0.17%), suicide attempt (0.08%), mood altered (0.08%) and depression suicidal (0.08%). **c**. Cardiac disorders in patients with breast cancer treated with ZA, included tachycardia (3.04%), cardiac failure congestive (1.35%), arrhythmia (1.01%), palpitations (1.01%), left ventricular dysfunction (0.76%), cardiomegaly (0.59%), cardiac disorder (0.51%), cardiac failure (0.51%), myocardial infarction (0.42%), atrial fibrillation (0.34%), heart rate decreased (0.34%), cardio-respiratory arrest (0.17%), endometrial hypertrophy (0.17%), cardiovascular somatic symptom disorder (0.08%), cardiovascular disorder (0.08%), and cardiac discomfort (0.08%).

**TABLE 8:** Top 6 AEs not registered in specification related to prostate cancer treatment.

Indication	Denosumab (n = 1225)		Zoledronic acid (n = 916)	
	AEs	N (%)	AEs	N (%)
**Prostate cancer**	Death	109 (8.9)	Death	315 (34.4)
	Prostate cancer metastatic	20 (1.6)	General physical health deterioration	182 (19.9)
	Renal impairment ^**a**^	26 (1.7)	Hemoglobin decreased	173 (18.9)
	Emotional disorder ^**b**^	20 (1.6)	Prostatic specific antigen increased	169 (18.4)
	Cardiac disorders	16 (1.3)	Cardiac disorders	125 (13.6)
	Disease progression	13 (1.1)	Malaise	113 (12.3)

**Note: a**. Renal impairment included renal failure (0.82%), renal impairment (0.33%), blood creatinine increased (0.33%), acute kidney injury (0.24%), renal failure acute (0.16%), renal disorder (0.16%), and blood creatinine abnormal (0.08%). **b**. Emotional disorder in patients with prostate cancer treated with Dmab included nervousness (0.08%), anxiety (0.33%), abasia (0.16%), irritability (0.16%), restlessness (0.16%), aggression (0.16%), agitation (0.08%), anger (0.08%), anxiety (0.08%), depressed mood (0.08%), depression (0.08%), and hallucination (0.08%).

In prostate cancer treatment, the top AEs associated with Dmab were death (8.9%), prostate cancer metastatic (1.6%), and renal impairment (1.7%), while for ZA, the most common AEs were death (34.4%), general physical health deterioration (19.9%), and hemoglobin decreased (18.9%). Additionally, ZA was also associated with increased prostatic specific antigen (18.4%) and cardiac disorders (13.6%), while Dmab was associated with emotional disorder (1.6%) and cardiac disorders (1.3%).

## 4 Discussion

### 4.1 Descriptive analysis

In this study, we performed a pharmacovigilance analysis using FAERS to investigate suspected AEs and off-label uses associated with Dmab and ZA. The data covers a substantial timeframe from 2004 to 2022, during which these two medications were administered in clinical practice at different time periods. Notably, the reporting rate for AEs can differ not only among various drugs but also for the same drug as time progresses (Moore et al., 2007; Alatawi and Hansen, 2017). Additionally, media attention, regulatory measures, Risk Evaluation and Mitigation Strategy, new indications, formulation changes, or shifts in marketing approaches can impact the adverse events profiles (Chhabra et al., 2013). Furthermore, both drug reporting trends exhibit a Weber-like effect (Hoffman et al., 2014; Noguchi et al., 2021), where AEs increase prior to marketing approval and subsequently decrease. Consequently, these variations in usage timelines may have led to different adverse event profiles, potentially impacting the results of our data analysis.

Nonetheless, the current understanding of these drugs is not yet fully comprehensive, and many AEs still require adequate attention. To better understand the AE profile of these drugs, it is recommended to collect as much clinical data as possible and conduct more in-depth analysis and evaluation.

### 4.2 AE signals with higher ROR values

The most frequent AEs of Dmab were death, osteonecrosis of jaw, back pain, tooth disorder, bone pain and hypocalcaemia and those for ZA were osteonecrosis, death, pain, osteonecrosis of jaw, and tooth extraction. The AEs identified in this analysis were generally in line with the known AEs of these drugs, indicating the validity of the study and suggesting that the findings may accurately reflect real-world clinical practices.

It is known that Dmab and ZA share many similar AEs. In our study, we conducted a comparative analysis to assess the signal strength of AEs between these two drugs. Among the signals of Dmab stronger than ZA (*d > 20*), the significant signals were exostosis of jaw, atypical fracture, and atypical femur fracture, suggesting that Dmab may be more prone to these AEs than ZA. Exostosis of jaw may be associated with the widely recognized osteonecrosis of the jaw (ONJ), which is a rare but serious side effect of anti-bone resorption inhibitors. Although a study demonstrated that patients with bone metastases treated with Dmab or ZA had similar incidences of ONJ (Nicolatou-Galitis et al., 2019), a meta-analysis of patients with solid tumors found that the use of Dmab was linked to a significantly higher risk of ONJ compared to ZA (Boquete-Castro et al., 2016). It is important to note that the incidence of ONJ may also be related to the dosage and duration of drug exposure (Khan et al., 2015). Thus, long-term and high-dose use of Dmab or ZA requires vigilance against ONJ. In contrast, the signals of ZA stronger than Dmab (*d > 50*) were mostly related to oral problems, which may also have potential implications for ONJ. Regular dental examinations should be conducted when using Dmab and ZA.

### 4.3 Off-label use with higher frequency in the database

Dmab and ZA, have been approved for preventing bone metastases associated with solid tumors. However, our research has found that these drugs are also frequently used in bone metastasis-free cancer. It should be emphasized that in some reports, cases of non-bone metastatic cancers may have been reported ambiguously without clear indication of the presence or absence of bone metastasis, thereby posing a limitation to the study. The theory of cancer treatment may primarily base on preventing cancer treatment-induced bone loss. Furthermore, some studies have shown that both drugs have potential anti-cancer properties (Dedes et al., 2012; Ubellacker et al., 2017; de Groot et al., 2018), but whether they have a positive effect on fighting cancer remains a matter of debate.

Postmenopausal women with breast cancer have a higher risk of osteoporosis due to the decrease in estrogen, as well as the effects of chemotherapy, radiotherapy, endocrine therapy, and the tumor itself (Guise, 2006; Chen et al., 2009; Gralow et al., 2013; Shapiro, 2020). Endocrine therapies such as tamoxifen and aromatase inhibitors have been shown to increase bone loss or fracture risk in both pre- and postmenopausal women with early-stage breast cancer (Powles et al., 1996; Sverrisdóttir et al., 2004; Aihara et al., 2010; Zaman et al., 2012; Tseng et al., 2018). Dmab 60 mg is approved for aromatase inhibitor-induced bone loss in women with breast cancer regardless of whether there is bone metastasis, while ZA did not receive such approval. Interestingly, ZA is reported to be used for preventing bone loss or decreasing fracture in premenopausal women with breast cancer (Gnant et al., 2015; Wilson et al., 2018). There is no clinical evidence that Dmab is suitable for use this population. Evidence suggests that Dmab or ZA could be applied as adjuvant therapy to improve bone density in postmenopausal women with early-stage breast cancer (Brufsky et al., 2009; Waqas et al., 2021). Note that one phase 3 trial shows that Dmab did not improve disease-related outcomes and did not support a role as an antitumor agent in early-stage breast cancer for women with high-risk early breast cancer, in addition to the benefits of delaying cancer bone-related events (Coleman et al., 2020).

Antihormonal treatments for prostate cancer can also cause bone loss. The FDA has approved Dmab (60 mg) for the treatment of bone loss or preventing fracture in non-metastatic prostate cancer, while ZA currently lacks FDA approval. Several small randomized trials have shown that bisphosphonates can increase BMD in patients with non-metastatic prostate cancer (Smith et al., 2001; Smith et al., 2003; Klotz et al., 2013). Note that no benefit has been shown among bisphosphonates in preventing fractures among patients with nonmetastatic prostate cancer (Strum et al., 2018).

As for the treatment of osteoarthritis (OA), a study in rabbits with experimental knee osteoarthritis showed that ZA had protective effect on articular cartilage and subchondral bone (She et al., 2017). An initial trial showed that ZA may be effective in treating osteoarthritis (Aitken et al., 2018). However, we have not yet found strong evidence that osteoarthritis can benefit from ZA. Markers of bone turnover are increased in patients with progressive OA, similar to those in patients with postmenopausal osteoporosis (Bingham et al., 2006). Based on that mechanism, ZA may have a prospective benefit for osteoarthritis. Regarding Dmab, it has rarely been reported in osteoarthritis, but evidence suggests that Dmab may be a potential new therapeutic option for treating rheumatoid arthritis (Hu et al., 2021; Tanaka et al., 2021).

In conclusion, mining new indications from the database has the potential to expand drug application range, promote drug research and development, and improve clinical practice. However, it is crucial to conduct further real-world research to validate these new indications and ultimately benefit patients.

### 4.4 AEs with higher report frequency in breast cancer and prostate cancer

According to the reports, disease progression was observed more frequently in the treatment of breast cancer and prostate cancer with Dmab or ZA. However, current evidence does not establish a definitive link between tumor progression and drug exposure. Our study also found a high frequency of neutropenia among breast cancer patients treated with Dmab, which is consistent with reports of neutropenia in a phase III study of multiple myeloma patients treated with both Dmab and ZA (Raje et al., 2018). Mental problems were also reported in breast cancer patients treated with either drug, although drug-induced mental disorders on Dmab or ZA are currently poorly documented. A case report indicated that extreme anxiety and hypocalcemia after denosumab treatment for cancer-related bone metastasis may have contributed to depressive mood (Lin et al., 2015). Although atrial fibrillation is a known AE to Dmab, our study also found a high frequency of heart problems in ZA-treated patients. Previous studies have reported an increased rate of heart failure in zoledronate-treated patients (Black et al., 2007; Rubin et al., 2020), suggesting that more clinical trials are needed to confirm the safety of ZA. Renal toxicity is a potential AE of ZA treatment, although Dmab is considered relatively safe for the kidneys. However, renal toxicity has been observed in the treatment of multiple myeloma using Dmab (Raje et al., 2018). From the pharmacokinetics perspective, Dmab is not metabolized by the kidneys and theoretically has minimal damage to the kidneys, it is still relatively safe.

## 5 Limitation

It's important to acknowledge several limitations that raise questions about its direct real-world applicability. Looking at the FAERS database, there are several aspects to consider. First, the cases registered in spontaneous reporting systems are only those of drug-induced AEs, not the total number of patients treated with the drugs (Noguchi et al., 2021; Marwitz and Noureldin, 2022; Crisafulli et al., 2023), making it difficult to compare the incidence of AEs between Dmab and ZA. Second, some reports may lack important information such as outcome, indication, dose, age, and sex (Shao et al., 2021; Tang et al., 2022), leading to potential bias in the analysis. Additionally, the accuracy of the data may be compromised due to the involvement of non-professional reporters (Bian et al., 2021) and the absence of a standardized reporting format. Furthermore, it should be noted that some reported AEs may actually be different manifestations of the same underlying condition, such as jaw exostosis, jaw abscess, and exposed bone in jaw, all of which may be related to osteonecrosis of the jaw. Although the study has attempted to integrate such AEs, there is still a possibility of some omissions. In addition, the presence of “notoriety effects” leading to increased reporting of specific adverse events can limit the study due to potential underestimation (Pariente et al., 2007; Noguchi et al., 2021).

Regarding disproportionality analysis, it solely represents statistical correlation between drugs and AEs and do not permit the establishment of causal associations between reported AEs and specific medications (Abe et al., 2015; Michel et al., 2017). Furthermore, it comes with the limitation of false-positive signals and suffers from the limitation of lower specificity (Noguchi et al., 2021).

Also, the study’s failure to compare the impact of different specifications on indications may lead to incomplete evaluation of the drugs’ safety and efficacy. Additionally, it solely focuses on potential off-label use, neglecting over-the-label use of different specifications and their safety profiles, potentially overlooking certain safety issues and differences in effectiveness. Furthermore, the article may be affected by selection bias in data or inadequate analysis methods, which may impact the accuracy of drug evaluation and conclusions. The article may also not fully consider other factors affecting drug use, such as individual differences among patients, comorbidities, or the influence of other drugs.

However, the FAERS database gathers AE reports associated with drugs and therapeutic biologic products, which is a valuable resource to identify potential safety issues. Despite the aforementioned limitations, disproportionality analysis is now a validated method in the field of drug safety research and surveillance (Montastruc et al., 2011). It has high sensitivity and could serve as a foundation for generating hypotheses in future research endeavors (Abe et al., 2015; Noguchi et al., 2021; Crisafulli et al., 2023). Moreover, it can offer further insights into the influence of regulatory and policy decisions on AE reporting (Marwitz and Noureldin, 2022). Additionally, it's worth noting that there is a correlation between the risk of adverse reactions studied through meta-analysis and disproportionality analysis in many cases (Khouri et al., 2021).

## 6 Conclusion

In conclusion, our study found that both Dmab and ZA have similar trends in AE distribution. However, Dmab is statistically associated with a higher risk of jaw exostosis and atypical femur fractures, while ZA has a statistical link to more oral problems. It is important to note that both drugs have potential applications beyond their approved indications, particularly in the treatment of various cancers and osteoarthritis, and some new AEs may come with those off-label use, including mental health disorders, neutropenia and kidney damage, and heart problems. Given the correlation between the analysis results from spontaneous reporting systems databases and clinical safety studies (Khouri et al., 2021)**,** our findings highlight the importance of safety monitoring when using Dmab and ZA off-label. Moreover, considering the limited research focused on this specific aspect, our study may serve as a reference point for future investigations, contributing to drug safety vigilance efforts. Finally, due to the inherent limitations of spontaneous reporting databases, which inevitably contain potential biases, there is an urgent need for well-designed comparative safety studies to validate these findings.

## Data Availability

The datasets presented in this study can be found in online repositories. The names of the repository/repositories and accession number(s) can be found below: http://openvigil.sourceforge.net/.
